# Supplementary material for: A survey of experts to identify methods to detect problematic studies: Stage 1 of the INSPECT-SR Project
Source: medRxiv. 2024 Mar 25:2024.03.18.24304479. Preprint. [Version 2] doi: 10.1101/2024.03.18.24304479 (PMC10996715; doi:10.1101/2024.03.18.24304479)
Supplement: Supplement 1 [file NIHPP2024.03.18.24304479v2-supplement-1.pdf]

SUPPLEMENTARY MATERIAL

Section A. List of comments.
Section B. Updated list of checks for problematic studies
Section C. Items excluded from the survey.

Section A. List of comments. Note that identifying information has been redacted.

Inspecting the results in the paper

Is the number of participant withdrawals compatible with the disease, age and timeline?	Good [authors' note: respondent wrote this for almost every question]
	Withdrawal rates between papers by the same team are often interesting: simulation can expose very unlikely common rates etc.
	What is the standard? It opens the door for guessing?
	and local context e.g. I dont think certain cultures have many withdrawals
	In many fields this is impossible to tell.
	Unclear
Are subgroup means incompatible with those for the whole cohort?	In some circumstances, rounding can cause subgroup means to not add up.
	What "means"?
	Beware of Simpson's paradox
	Are subgroup means *and variances*...
	This one is suddenly phrased as incompatible rather than compatible.
Are the reported summary data compatible with the reported range?	What does this mean?
	This criteria is highly suggestive of an error, but I fear that it has poor specificity for scientific misconduct. It is suggestive of very poor reporting.
	important
Are the summary outcome data	Would be covered by tests of dispersion above
	how many duplicates in a table count too :)

medRxiv preprint doi: https://doi.org/10.1101/2024.03.18.24304479; this version posted March 25, 2024. The copyright holder for this preprint (which was not certified by peer review) is the author/funder, who has granted medRxiv a license to display the preprint in perpetuity.
It is made available under a CC-BY 4.0 International license .

identical or nearly identical across study groups?	I think this is irrelevant.
	important
Are there any discrepancies between data reported in figures, tables and text?	including those in supplementary files?
	Or data in graphs are not given as numerical data anywhere in tables or text?
	Please, may you consider also dicrepancies between results section and abstract?
	Threshold should be identified.
	Quite frequent, not very specific
	not sure if that's a red flag for me due to printing/human error
	This one feels vague
Are any baseline data implausible with respect to magnitude or variance?	Please add prompt or signalling questions.
	important
	I am wondering if dividing this section into questions that a) require and b) don't require subject matter expertise in the research topic would be helpful? Some of these could be assessed by researchers and methodologists without knowing anything about the topic, others require intimate knowledge of the area under study.
Are any outcome data, including estimated treatment effects, implausible?	or too large ?
	Who can judge a result is implausible without replicating the trial in the same circumstances, territory, patients characteristics etc.
	Why "estimated" not "reported" or "calculated"?
	first you need to define what's implausible; secondly, I feel that 'fake' trials wouldn't go for implausible effects
	Without a range of definitions for what is covered by implausible such as which reference point, this will be very difficult to assess.
	Though I agree this is very important, this criterion seems like it is best evaluated by someone with some subject matter expertise in the area. Is there a way to make this more general? Maybe something like "How closely do the outcome data agree with treatment effects from other studies?"
Are any of the baseline data excessively similar between randomized	similar or different, these two ideas could be combined, so drop the row below on difference. It's about under- or over-dispersion
	also observing distribution plots of baseline p values. Are reported p values consistent with reported data
	need to take rounding into account (how many digits)

medRxiv preprint doi: https://doi.org/10.1101/2024.03.18.24304479; this version posted March 25, 2024. The copyright holder for this preprint (which was not certified by peer review) is the author/funder, who has granted medRxiv a license to display the preprint in perpetuity. It is made available under a CC-BY 4.0 International license .

groups?	Rounding has effect on this. Personal evaluation, unless the mean (SD) is reported to 4 decimal places, no method can account for rounding including Monte Carlo simulation.
	Though not recommended, p-values for demographics often reported. They must be uniform.
	What does "excessively similar". What if a trial is stratified?
	or excessively different
	I would specify "not explained by stratification of randomization on these covariates or randomization by minimization"
	I cannot find the reference quickly but XXXX use one of these for their papers, is that XXXX
	important, but needs accessible tool to perform that easily
	I love the inclusion of specific examples here - references would be helpful too for those not familiar
Are any of the baseline data excessively different between randomised groups?	Particularly, if the detected excessive difference is detected in prognostic covariates, this can indicate a manipulation.
	Is this a problem for a non-stratified variable?
	excessively similar more of a problem?
	In case of fully fabricated data, one can expect perfect data, fully compliant with what is expected in the ideal case. For the detection of fraud, poor inter-patient variability is a suggestive sign
Are there any discrepancies between the values for percentage and absolute change?	not completely clear to me
	What is "percentage change"? Do you mean "relative"?
	This item should be reformulated to make it more generic. "Are there any mathematical inconsistencies between summary statistics, such as an absolute difference of proportions inconsistent with the two given proportions"
	Subsitute "relative" for "percentage" for clarity and avoid limiting the scope
Are there any discrepancies between reported data and participant inclusion criteria?	Threshold should be identified.
Are the variances	What "variances"?

medRxiv preprint doi: https://doi.org/10.1101/2024.03.18.24304479; this version posted March 25, 2024. The copyright holder for this preprint (which was not certified by peer review) is the author/funder, who has granted medRxiv a license to display the preprint in perpetuity.
It is made available under a CC-BY 4.0 International license .

in biological variables surprisingly consistent over time?	'surprisingly' is not an objective word.
	could we better define "surprisingly consistent?"
Are correct units reported?	Is this typographical?
	In my opinion, this has very poor specificity. This problem is mostly due to poor reporting.
	Love this
Are numbers of participants correct and consistent throughout the publication?	How would you know that they are "correct"?
	Including flowchart of how they arrived at that number. Could be cross-referenced to other studies that claim to use the same data.
	Often, there are errors at this level because authors do not explain well.
Are calculations of proportions and percentages correct?	Relates to missing data point below. Sometimes ITT is used as denominator, ignoring missing data.
	Is this typographical?
	This is predicated on knowing the denominator/ analysis set total, usually from Consort flowchart.
	Problematic but not very specific of misconduct in my opinion. This is suggestive of copy errors and statistics performed by non-statistician.
	important and easy to do
Are results internally consistent (for example, are there more births than pregnancies)?	Needs question on textual discrepancies, e.g. a trial for men that provides that gives results for women, or menopause dates in baseline data
	What about twins?
	Is this phrased in the "red flag" manner on purpose? I think if you ask "is it consistent", the example should be consistent, and not it is not. There should be more pregnancies than live births.
	important
	I see where this example is going, but a small amount of discrepancy would be OK due to stillbirths and multiple gestations.
Are non-first digits compatible	Not sure what is meant byt this.
	wording is unclear, I'm not sure what this means

medRxiv preprint doi: https://doi.org/10.1101/2024.03.18.24304479; this version posted March 25, 2024. The copyright holder for this preprint (which was not certified by peer review) is the author/funder, who has granted medRxiv a license to display the preprint in perpetuity.
It is made available under a CC-BY 4.0 International license .

with a genuine measurement process?	Is this Benford's law? Definitely worth using
	Is this, specifically, even digits?
	What does this mean?
	I think this phrasing confuses me quite a lot. Do you mean "are digits (other than the first) repeated in a manner that is to be expected?"
	This feels too vague to me - are you looking specifically for randomly distributed terminal digits as indicative of a low likelihood of bias in measurement? Making this a little clearer would help
Are the variances of integer data possible?	Needs explanation
	GRIM, GRIMMER I figure?
	Not something I've looked at.
	What integer data?
	important
	I would love a reference or tutorial on this
Are the means of integer data possible?	Why possible, not plausible? What integer data?
	Maybe this one could be combined with the previous question asking about variances??
Are data simulated from reported summary statistics plausible?	comparing tables across hunders (or thousands) of papers will be computationally challenging
	This is helpful but not absolute. Consider that the generated distributions from the age variable with a mean (SD) of 31.3 (3.5) represent any potential distribution of the variable with a mean between 31.25 and 31.35 and an SD between 3.45 and 3.55. This could result in billions of possible distributions, one of them represent the study arm. Even these kind of extensive simulation cannot be done in regular computers.
	What data will be "simulated"?
	Not sure what is meant here?
	I would remove the "simulated" word in this sentences
	This requires subject matter expertise that I don't have and woud love some references or information on this.
Are differences in variances in baseline variables between	What variances?
	Beware of Bartlett/F tests. They heavily rely on the normality assumption. If they are very significant (p<1e-6), they still may be useful. P-values too close to zero and too close to 1 are equally interesting (too much or not enough heterogeneity

medRxiv preprint doi: https://doi.org/10.1101/2024.03.18.24304479; this version posted March 25, 2024. The copyright holder for this preprint (which was not certified by peer review) is the author/funder, who has granted medRxiv a license to display the preprint in perpetuity.
It is made available under a CC-BY 4.0 International license .

randomised groups plausible(using summary data, e.g. F test, Bartlett)?	of variances)
	important, but needs accessible tool to perform that easily
	Love this
Are coefficients of variation plausible?	No something I've looked at.
	What coefficients?
	Redundant with "Are reported summary statistics plausible"
	some detail on how to determine this would be useful
Is the amount of missing data plausible?	and clearly reported ?
	This varies depending on how each centre counsels its patients.
	maybe reword to include - is there implausible lack of missing data
	Or investigated at all.
	Baseline characteristics, intermediate outcomes and final outcomes could have different proportions of missing data.
	Would this link to risk of bias rating - e.g., attrition
Are the results substantially divergent from the results of multiple other studies in meta-analysis	What meta-analysis?
	Will depend on the study inclusion criteria for the underpinning system review.
	I would reformulate : "implausibly divergent from the results [...] taking in account the methodology bias". Indeed, major divergence is expected with a very poor methodology without misconduct.
Is there heterogeneity across studies in degree of imbalance in baseline characteristics (in meta-analysis)	Why does this relate to imbalance, rather than the characteristics themselves?
	Is heterogeneity reasonably considered in the conclusion. I'm thinking about basing results on the prediction interval in random effect meta-analysis.
	Will depend on the study inclusion criteria for the underpinning system review.
	It would only diagnose an overall problem of the literature, that may be partly due to poor randomization schemes. Outliers in imbalance (very large or very small) are more interesting.
	I do not really understand this one. Is that a sign of fraud? Much less intuitive to me.
	This wouldn't be surprising, would it?

medRxiv preprint doi: https://doi.org/10.1101/2024.03.18.24304479; this version posted March 25, 2024. The copyright holder for this preprint (which was not certified by peer review) is the author/funder, who has granted medRxiv a license to display the preprint in perpetuity.
It is made available under a CC-BY 4.0 International license .

Inspecting the research team

Have the data been published elsewhere by the research team?	But excluding conference presentations?
	Does this need a qualifier, like 'without being acknowledged' - or does this make it too similar to the question above? Is publication of data elsewhere sufficient as a red flag?
	If for example, the data is re-published and the authors are transparent about this, would this item be used to identify the study as not problematic?
	Checking self-citation
	not always easy to ascertain
	While I agree this is important, it would be difficult to verify.
Is any duplicate reporting acknowledged or explained?	Not sure why this is additional to point below.
	Duplicates within the paper or between papers?
	golden dust...
	"In one review, we included a trial by XXX. We found this study has multiple publications which is fine, but one of these was retracted and was identical to another publication. This issue was not explained by the author team in their publications: [author note: details of publications provided]"
Are duplicate-reported data consistent between publications?	or " is there a high rate of identical and / or highly similar data across asrtic;es from the same team
	the meaning of this is unclear to me; do you mean expressly duplicated data that the authors acknowledge has been published in prior papers?
Are relevant methods consistent between publications?	Again this is too vague. Do you mean that it would be strange if a research team used one sort of standard treatment for one study but a different one in another, when if it was at the same hospital you would expect the standard care arm to be the same?
	'relevant' might need explanation?
	Between what publications?
	"of the same database ?"
	of whom?

medRxiv preprint doi: https://doi.org/10.1101/2024.03.18.24304479; this version posted March 25, 2024. The copyright holder for this preprint (which was not certified by peer review) is the author/funder, who has granted medRxiv a license to display the preprint in perpetuity.
It is made available under a CC-BY 4.0 International license .

Is there evidence of duplication of figures?	is this an authorship thing?
	this seems more about specifics than the team?
	be explicit: numbers or pictures?
	Figures as in graphs and also where images (radiological, histological etc) are presented.
	How does this compare with question above "Is there evidence of manipulation and duplication of the images".
	This seems like it should go under the "inspecting the results" section
	Or tables
Does consideration of other studies from members of the research team highlight causes for concern?	I usually take one study at a time and would not have the time to do this.
	How does this compare to the question above about retractions for other studies of the author team?
Is the distribution of non-first digits in manuscripts from one author compatible with a genuine measurement process?	Love this - but please add an explainer otherwise people will just start using similar thinking to 'P values in Table 1 = NS'
	Wording is unclear, I don't understand what the distribution of non-first digits would indicate.
	not clear why this would be limited to papers by single authors
	the validity of this method can be questioned
	I evaluated more than 2000 values in summary statistics for a group of authors with known integrity and I found then not compatible with genuine process. Perhaps, rounding has a role.
	why have you written 'non-first' rather than 'final' or 'last digits' in numerical data? (Presumably you don't mean to include middle digits of 3 digit numbers?). Why have you included the phrase 'manuscripts from one author' rather than 'in this paper'? Could consider adding 'or does it raise the suspicion of data falsification'?
	What does this mean?
	this isn't really inspecting the team?
	This would not be feasible when one author has a big number of publications.
	Same as above
	Add 'multiple' manuscripts here to clearly distinguish from earlier item?

medRxiv preprint doi: https://doi.org/10.1101/2024.03.18.24304479; this version posted March 25, 2024. The copyright holder for this preprint (which was not certified by peer review) is the author/funder, who has granted medRxiv a license to display the preprint in perpetuity.
It is made available under a CC-BY 4.0 International license .

	Unclear
	I love this one - though specifying terminal digit analysis may be more user-friendly for reviewers. And more specifically, I think looking for a signal of overrepresented 0's and 5's in the last digit place would be easiest to do.
Is the standard deviation of summary statistics in multiple studies by same authors plausible (when compared to simulated or bootstrapped data?)	I'm not sure what the "standard deviation of summary statistics" means. Does it refer to SDs that appear in the summary statistics, or the SD of the statistics themselves (as calculated by the reader)?
	Even within the same group, eligibility requirements and recruitment circumstances may vary. In developing nations, for instance, the features of recruiting patients from poor villages vary from those in other areas. In the village, women marry at a younger age and are more likely to be overweight due to their lack of full-time work and physical activity. Also as an illustration, in one of my trials, recruitment was primarily dependent on two referring satellites (one in the village and the other in the main city). The majority of women recruited from the clinic in the village were near to 22 years old, whereas those recruited from the clinic in the city were close to 30 or older. This gives the data on age a bimodal look, which is not quite common in RCTs. This will not be known until each scenario is explained individually. Although the inclusion criteria and authors are the same, the distribution of patients' age recruited for the other concurrent trial with the same inclusion criteria is different. Each trial should be evaluated in light of its unique circumstances.
	by 'summary statistics' do you mean 'baseline patient characteristics'? Could this be relevant even if identified in one study?
	Why in multiple studies?
	Be very cautious with this item... Summary statistics (e.g. mean age) are expected to be very different in the multiple studies of the same author if there are different inclusion criteria. If would restrict this criteria to "Several articles on very similar populations, with similar inclusion criteria, within the same centers, show a major inconsistency in some summary statistics that ought to be similar".
	As a reviewer, I would love to have some detail on how to do this, and some references showing appropriate use
Do all authors meet	how would you ever know?

medRxiv preprint doi: https://doi.org/10.1101/2024.03.18.24304479; this version posted March 25, 2024. The copyright holder for this preprint (which was not certified by peer review) is the author/funder, who has granted medRxiv a license to display the preprint in perpetuity.
It is made available under a CC-BY 4.0 International license .

criteria for authorship?	very hard to judge. I think contributor statements are often fictional, especially when there are bullies on the team
	how will you know that?
	This, and some other items, would/should be automated in the submission processess?
	Maybe unreasonable number of authors?
	Plausible number of authors too.
	Very difficult to gage, especially in this day and age of medical research
	not always possible to ascertain
	How would you go about checking this?
	as defined by ICJME, presumably?
Are contributorship statements present?	I'd combine this with the "complete" criterion as "present and complete"
	could be merged with first statement in this section
	Do you intend to word the questions consistently such that 'yes' and 'no' and consistently indicative of 'suspicious' eg if 'yes' = suspicious then this questions should be 'are contributorship statements lacking'
	Important!
	Is this more an issue for journal styles than problematic studies?
	see comments above
	Not relevant in my opinion. This is more dependent of the editor than of authors.
	journal specific section
Are contributorship statements complete?	As these are rarely required by journals, this might be a difficult domain to assess
	Why not ask this next to the earlier question on contributorship? Is it a reflection on the journal?
	This seems include the following "Are contributorship statements present?" - suggest merging them
	not sure if this is available in all journals
	How does this compare with the question above "Are contributorship statements present?" Could the two be merged?
Is authorship of related papers consistent?	I think this needs a bit of clarification. Do you mean if multiple papers arise from a single RCT, we would tend to expect high overlap of authors?

medRxiv preprint doi: https://doi.org/10.1101/2024.03.18.24304479; this version posted March 25, 2024. The copyright holder for this preprint (which was not certified by peer review) is the author/funder, who has granted medRxiv a license to display the preprint in perpetuity.
It is made available under a CC-BY 4.0 International license .

	Not clear to me.
	Is this considered a red flag?
	not sure if I understand the question
Can co-authors attest to the reliability of the paper?	How can judge? It opens the door for personal opinions and conflict of interest. every single publication should be judged separately. Authors may have serious honest errors that can be misclassified as integrity issues. If they progress and learn to do better research, they should be encouraged and their good publications should be seen as good.
	Can only obtain by asking them, and often there's no email for non-corresponding authors.
	when joined?
	Does this involve contacting the co-authors to ask?
Have other studies from the author team been retracted, or do they have expressions of concern, relevant post-publication amendment, or critical retraction	Each trial should stand by itself.
	Be careful about this. As a cautionary tale, see Peto R, et al. The trials of Dr Bernard Fisher: a European perspective on an American episode. Controlled Clinical Trials 1997;18:1-13.
	Post-publication amendments seem not a problem as most of them were due to unintentional errors, usually minor. PubPeer comments can be muddy. Anyone could post something, including those who have no expertise at all or have unverified intentions.
	definitely worth being aware of
	scite.ai can possibly help with this
	This is an example of a good criterion in my view because it provides specific places reviewers should look for problems (retraction watch, pubpeer)
Are the authors on staff of institutions they list?	This can be very difficult to ascertsin, especially years after publication of the article.
	Do authors have institutional email addresses?
	Not sure if this will be relevant for all countries. For example medical residents are not in thehospital's website
	this seems very useful.
	Does this discrimate against, for example, PPI?
	This seems like a typo? Do you mean, are they employed by whom they say they are employed?
	not always easy to ascertain

medRxiv preprint doi: https://doi.org/10.1101/2024.03.18.24304479; this version posted March 25, 2024. The copyright holder for this preprint (which was not certified by peer review) is the author/funder, who has granted medRxiv a license to display the preprint in perpetuity.
It is made available under a CC-BY 4.0 International license .

	How should we verify this?
Do any authors have a professorial title but no other publications on PubMed?	Why limit to professorial?
	interesting. I think only some journals include titles
	pubmed limited in coverage
	Not a check that I've done.
Does the statistics methods section use generic language, suggesting lack of expert statistical input?	Suggest things to look out for e.g. 'Begger's Test'
	will catch lots of bad practice rather than fraud, but that's still worthwhile
	Or might it suggest good, plain language writing?
	Nice. https://journals.plos.org/plosone/article?id=10.1371/journal.pone.0264360
	Is this misconduct or poor reporting? Moreover, this item should not be in the section "inspecting the research team".
	I do not think generic language suggests problems. It is more important to assess the content in statistics methods.
	may be penalising
	This is almost every paper!
	This is tricky because I wouldn't want simplicity to trigger suspicion for lack of specificity. Maybe rephrase to something like "Does the statical methods section use language that is too vague or general to permit replication?"

Inspecting conduct, governance and transparency

Is the volume of work reported by research group plausible, including that indicated by concurrent studies from the same group?	Consider adding a clarification after 'volume' (e.g. number of publications in a specified amount of time)
	This cannot be determined by guesswork and varies by country and facility. For some facilities, it is possible to enrol thouthands of individuals within a year, whereas in developed nations, a trial with 1,000 participants may require 25 participating sites and five years to complete. This criterion should not be applied to other communities (one-team-centrism [western-centrism] should not apply to other researchers from different communities). A one-by-one judgement is likely the most effective.
	important but seldom looked at

medRxiv preprint doi: https://doi.org/10.1101/2024.03.18.24304479; this version posted March 25, 2024. The copyright holder for this preprint (which was not certified by peer review) is the author/funder, who has granted medRxiv a license to display the preprint in perpetuity.
It is made available under a CC-BY 4.0 International license .

	Unless you have a platform trial situation.
	Reword: Is the volume of work and concurrent studies reported by the research group plausible?
	Maybe include suggestions for how to assess this - e.g. using the author field to search pubmed for first and last author?
Is the reported staffing adequate for the study conduct as reported?	Staffing might only be deduced from authorship of paper
	Do studies report staffing?
	Unlikely to be reported
	I do not understand this one. Do you mean that every role is filled such that it is plausible the study was conducted? For instance, a multicenter study with three authors seems unlikely (collaborators would want to be on the paper)
	I noticed that for some studies we identified for a review, many RCTs had only one author. This raises our concern about if the so-called trial is a true trial.
	This feels vague to me and I'm not sure how someone would be able to evaluate this for areas outside their subject matter expertise unless it was glaringly obvious (e.g., 3 people cannot conduct a multinational, multicenter RCT)
Is the recruitment of participants plausible within the stated time frame for the research?	Who can judge?
	sometimes time frame is not mentioned
	Brilliant one this
	this is what we looked at
	Are there any known benchmarks for this? Maybe we should develop some - could be an easy and cool paper.
Is the recruitment of participants plausible considering the epidemiology of the disease in the area of the study location?	Consider replacing disease with 'condition'. Not all trials focus on people with a disease.
	And size of research institution
	I think it will be hard to decide on the wording for a lot of these. Is it plausible? Yes. Is it likely? No. If there are lots of "unlikely" answers then they add together to a problem...
	maybe include details eg are there implausibly large numbers of participants recruited for a rare disease, or an implausibly small number recruited for a common disease and a well-resourced research study

medRxiv preprint doi: https://doi.org/10.1101/2024.03.18.24304479; this version posted March 25, 2024. The copyright holder for this preprint (which was not certified by peer review) is the author/funder, who has granted medRxiv a license to display the preprint in perpetuity.
It is made available under a CC-BY 4.0 International license .

	Developing a rule-of-thumb would be cool for this. For example, for a disease with an estimated prevalence of X per 100,000 people, a recruitment rate above Y is incompatible with that prevalence.
Is the interval between study completion and manuscript submission plausible?	Important point
	Important!
	another investigated aspect
Could the study plausibly be completed as described?	This needs an explanatory note. Does this refer to studies being conducted rather than completed?
	too vague?
	This looks vague.
	I think this might be a rather subjective question
	This feels too vague
Are the study methods plausible, at the location specified?	Please give an example or further prompt/signalling questions.
	Who can judge except the authors or their institute?
	Subjective unless you have experience in the setting
	Maybe a few examples between parentheses would help : (number and nature of examinations and interventions performed)
	another subjective question, picking only on extreme cases
Are the locations where the research took place specified, and is this information plausible?	What is this? Are the locations plausible? What does that men?
Is a funding source reported?	or funding sources, would it have provided sufficient funds for conducting the research
	Do we know that this is a risk of fraud though? Not having funding?
	The way this is posed could lead to ambiguity. Would it be better to distiguish cases in which funding source is not reported (because there was no funding source) and those in which funding is not discussed at all?
	I noticed that regarding the true or false of Chinese trials, a report mentioned that an RCT with a clear funding source statement could be true.
	Unlike some of the other categories, this one is an objective yes/no - easy for reviewers to assess.

medRxiv preprint doi: https://doi.org/10.1101/2024.03.18.24304479; this version posted March 25, 2024. The copyright holder for this preprint (which was not certified by peer review) is the author/funder, who has granted medRxiv a license to display the preprint in perpetuity.
It is made available under a CC-BY 4.0 International license .

Has the study been prospectively registered?	Needs a separate question about retrospective registration
	And Perhaps if the results are reported withnin a certain timeframe from reporting of study is complete (e.g. legal limits)
	ID of registration
	Would this call RECOVERY into question?
	Consider adding a year limit when prospective registration becomes mandatory (e.g. after 2010)
	Only applies to trials.
	in our case study was "prospectively reported2 on NCTN yet questionable
	Reword: Was the study prospectively measured?
	I wonder whether you could also ask for the reporting of the results on the trial registry (over and above registration)
	Adequate prospective registration is not happening for a lot of legitimate trials, so I am not sure this is relevant.
	This may require going to the individual registries to verify the date
Are details such as dates and study methods in the publication consistent with those in the registration documents? -	The 'historical versions' facility in ClinicalTrials.gov is very useful to trace the timeline of changes.
	might be one of the 1st things to check?
	study locations, heirarchy of outcomes, randomization methods, sample size
	This could be a multipart question.
	another investigated aspect
Is there evidence that the work has been approved by a specific, recognized committee? (ethics)	Evidence other than their own claims?
	recognised by whom?
	not sure if lack of this info would be an immediate red flag... some journals give very little space to describe methods
	Another specific yes/no question, which is great - but I wonder if the wording could be improved, to something like "Have the authors indicated that the work has been reviewed and approved by an instituitonal review board or ethics committee?"
Are there any concerns about unethical practice?	Do not make this yes no - suggest using 'Describe any concerns....'
	concerns by whom?
	vague question

medRxiv preprint doi: https://doi.org/10.1101/2024.03.18.24304479; this version posted March 25, 2024. The copyright holder for this preprint (which was not certified by peer review) is the author/funder, who has granted medRxiv a license to display the preprint in perpetuity.
It is made available under a CC-BY 4.0 International license .

	This feels like it could encompass problems more broadly than just research misconduct.
Is the grant funding number identical to the number in unrelated studies?	good, this should be expanded to ethics too, see where multiple unrelated studies have identical issues
	Again a flip in the phrasing (from "is it okay" to "is it not okay"), because I think you mean that this means they just copied in a random different grant number, right. Perhaps "is the grant funding number unique or related to similar studies?"
	there are typos in NCTN numbers not to mention grant numbers...
	This needs some nuance
	How would a reviewer know this?
Are the data publically available?	Please clarify if you mean data available outside the study publication.
	I sometimes wonder if a weird excuse not to provide the data is a bit of a flag too
	URL/Domain
	They almost never are so I dont see this being helpful at distinguishing between risky and other studies?
	Is this a good or a bad thing?
	"publicly"
	Consider clarifying what "data" means here, de-identified IPD or results published in trial registries.
	This is related to good conduct (open science), not misconduct. However, publically available data may help to detect some misconduct.
	This looks irrelevant.
	even with legit trials you might not have publically available data :(
	Or if not publicly assessible, then at least in a secure repository with proper links etc
Do the authors agree to share individual participant data?	Separate question about whether data is already provided needed
	Is this a good or a bad thing? Made up IPD can be shared more easily than real IPD
	Not specific of misconduct in my opinion. Authors rarely accept data sharing.
	answer as above

medRxiv preprint doi: https://doi.org/10.1101/2024.03.18.24304479; this version posted March 25, 2024. The copyright holder for this preprint (which was not certified by peer review) is the author/funder, who has granted medRxiv a license to display the preprint in perpetuity.
It is made available under a CC-BY 4.0 International license .

	This may not always be possible
Are additional patient data recorded in patient case records beyond what is reported in the paper?	Don't understand this question
	not clearly worded
	How could you possibly know this?
	Is this a good or a bad thing?
	Not sure this is relevant. Can you give an example here?
	Just a yes is not so informative. Is a list available with additional patient data ....
	not sure how would I be able to check that without direct contact with trialists
	Unclear
Do authors cooperate with requests for information?	there are legitimate reasons for not cooperating?
	Does the request originate from individuals who have no conflicts of interest or who do not focus their work on a certain geographical region?
	Is this a good or bad thing. Genuinely busy researchers might not have time?
	we had a person who replied to our email but the reply wasn't really helpful
Do authors provide satisfactory responses to requests?	What is the threshold of satisfactory response?
	Is this a good or bad thing. Genuinely busy researchers might not have time?
	answer above
	Do requests for study data merit a separate item?

medRxiv preprint doi: https://doi.org/10.1101/2024.03.18.24304479; this version posted March 25, 2024. The copyright holder for this preprint (which was not certified by peer review) is the author/funder, who has granted medRxiv a license to display the preprint in perpetuity.
It is made available under a CC-BY 4.0 International license .

Inspecting text and publication details

Is there evidence of copied work, such as duplicated or partially duplicated tables?	There should be a threshold before judgment.
	This involves assessment of other relevant papers. A bit of work.
	Or implausible distributions, e.g., over dispersion
	This may be beyond copied work: identical or similar (+/- 1 or 2) values in tables
	difficult to do / not feasible due to logistics (N included trials x number of authors x their other publications)
	meaning, from the same manuscript or from other manuscripts?
Is there evidence of text reuse (cutting and pasting text between papers), including text that is inconsistent with the study?	or image reuse
	suggest 'text or font that is inconsistent with the study'
	Some Cochrane reviews use standard text. Would this make them potentially "problematic"?
	I am more worried about numbers than words
	I have developed software that does this with a given set of PDFs. Working on performance improvements.
	Text reuse of methods of related apers may be appropriate
	Could this be automated? Plagiarism checks?
Are there typographical errors?	I think should be 'an unusually large number' of typos
	many publishers are increasingly limiting amount of copy editing offered, so I would not consider this a factor (unless in combination with a lot of other things, in which case typos are usually a moot issue anyway)
	clarify, not really seen this as a sign of fraud
	Could unfairly flag work from non-English speakers, but I agree that

medRxiv preprint doi: https://doi.org/10.1101/2024.03.18.24304479; this version posted March 25, 2024. The copyright holder for this preprint (which was not certified by peer review) is the author/funder, who has granted medRxiv a license to display the preprint in perpetuity.
It is made available under a CC-BY 4.0 International license .

	problematic papers are often sloppy
	not a reliable indicator
	how is this relevant for the integrity of the study?
	Typographical errors should not be a concern.
	I consider badly written manuscripts as indicate of a poorly conducted study.
	How might this identify studies that you define as "problematic" given that it is as much a role for the journal as the authors, and will bias against people who are writing in languages other than their main one
	Not relevant in my opinion, unless poor reporting is considered as scientific misconduct.
	This looks irrelevant.
	I don't see this as a red flag
	This would include many papers!
Is there evidence of automatically-generated text?	clarify please, is this tortured phrases?
	Not something I've seen / considered.
	Some Cochrane reviews use standard text. Would this make them potentially "problematic"?
	Specific examples? e.g. tortured synonyms
Has the study been retracted or does it have an expression of concern, a relevant post-publication amendment, a critical Retraction Watch or PubPeer comment or has been previously excluded from a systematic review?	amendments the same as corrections?
	PubPeer is potential good source of information but after validation and replication the comment provided
	I usually check PubPeer.
	Many studies are "excluded" from a systematic review for reasons that have nothing to do with being "problematic"
	Even though it is implied, you could also add letter to the editor as a specific point.
	I would add the words "due to concerns in scientific integrity" after "or has been previously excluded from a systematic review"
	important and possible to do
	see scite.ai's Reference Check feature.
	This issue is important to consider as I noted above.
Was the study	Please sign post to lists and explain what you mean by low quality journal.

medRxiv preprint doi: https://doi.org/10.1101/2024.03.18.24304479; this version posted March 25, 2024. The copyright holder for this preprint (which was not certified by peer review) is the author/funder, who has granted medRxiv a license to display the preprint in perpetuity.
It is made available under a CC-BY 4.0 International license .

published in journal from a list of predatory/ low quality journals?	Do you mean journal with substandard policies relating to editorial or peer review?
	...list of a journals with predatory practices ?
	predatory journal list/low quality is a criterium that often selects on global north publications as higher quality. what might be a more specific way of doing this?
	We may need to define low quality journals
	Or not PubMed/ Scopus indexed
	What list of predatory journals and how to define low quality journals?
	important aspect to check
	Journal quality could be a little disputed. It would be good to provide a list of what these journals might be.
	For some Chinese studies, this journal related factor could be considered in judging if an RCT is a true trial.
Is there evidence of manipulation or duplication of images?	"Duplication"?
	This question is a duplicate of a previous one

medRxiv preprint doi: https://doi.org/10.1101/2024.03.18.24304479; this version posted March 25, 2024. The copyright holder for this preprint (which was not certified by peer review) is the author/funder, who has granted medRxiv a license to display the preprint in perpetuity.
It is made available under a CC-BY 4.0 International license .

Inspecting individual participant data

Compare leading digits in individual participant data to Benford's Law	Be careful. Benford's Law (at least, the famous bit referring to the leading digits) often does not apply to scientific data because the range is (naturally) too constrained
	It is not useful for truncated data, e.g. age 18 to 40 in infertility trial. Clinical trials most probably has truncated data.
	What are "leading digits"?
	Not relevant and dangerous in my opinion. Benford's law is found when mixing statistics coming from very different horizons, but each variable will contain data that is very far from Benford's law, and it is expected that even when mixing all variables of a study they still do not follow Benford's law, although, that depends on the exact mix of variables available. For instance, the human weight has a first digit that is mainly between 5 and 9, very far from Benford's law. Overall, major departures from Benford's law are expected. Moreover, most variables have well known distributions (e.g. the human weight). Just check that distributions are consistent with the known distribution (mean, variance, skewness)!
	never heard of
Compare non-first digits in individual participant data to expected	Second digits may not be expected to be random. So check uniformity of 3rd digits (for minimum 3-digit data).
	What does this mean
	I think same as before? Perhaps harmonize the phrasing of those questions
	we don't do this
	This one feels like a repeat of an earlier question about terminal digit analysis
Compare distribution of leading digits in individual participant data between randomised arms	What are "leading digits"?
	Same as below (Benford's) but including a comparison? Unclear what that would add
	we don't do this
	I don't have the subject matter expertise to know what this is getting at - what would distribution of leading digits indicate? I'm guessing they should be similar between randomized arms?

medRxiv preprint doi: https://doi.org/10.1101/2024.03.18.24304479; this version posted March 25, 2024. The copyright holder for this preprint (which was not certified by peer review) is the author/funder, who has granted medRxiv a license to display the preprint in perpetuity.
It is made available under a CC-BY 4.0 International license .

Compare distribution of non-first digits in individual participant data between randomised arms	Can provide a false impression. For instance, if we are collecting FSH in a multicenter trial and one centre's device is miscalibrated and giving overreading, or under reading, this can produce contradictory results for non-first digits for authentic data. In addition, rounding will influence the analysis of non-first digits.
	What does this mean?
Comparing multiple versions of a spreadsheet containing study data for consistency	During data collection process, transcription errors are so common.
	What multiple versions?
	yes, basic consistency checks
Examining spreadsheet for formulae used to fabricate data	Has this happened? Are people really that stupid? That is insane.
	we don't do this formally but would be picked up when converting data to stata dta
Change global format to 'general' in Excel - calculated values display long strings of numbers to right of decimal place, fabricated values may not	It is amenable for transcription error and it is operator dependant.
	What does this mean?
	Interesting!
	we don't do this
Can the results in the paper be reproduced from the underlying dataset?	lack of reproducibility can come from many factors, not necessarily problematic
	Important. But how far do you go?
	This seems like a critically important question, but requires reviewers to essentially replicate the authors' entire analysis. Outside of special circumstances like reviewing cases of suspected fraud, I bet this is rarely (if ever) done.
Statistical test to compare variances in baseline variables between groups using IPD (Levene, Brown-Forsythe)	What variances?
	never heard of
Identifying inliers using singular value decomposition	I don't know what this is
	i don't know what this is!?
	What does this mean?
	i don't understand this one

medRxiv preprint doi: https://doi.org/10.1101/2024.03.18.24304479; this version posted March 25, 2024. The copyright holder for this preprint (which was not certified by peer review) is the author/funder, who has granted medRxiv a license to display the preprint in perpetuity.
It is made available under a CC-BY 4.0 International license .

	never heard of
	Unclear
Identifying inliers using Mahalanobis distance	I don't know what this is
	not familiar with this
	What does this mean?
	never heard of
	Unclear
Colour code values in Excel spreadsheet to highlight outlying values, patterns and repetition	no
	Why?
	we don't do this
	Unclear
Plot column values in order provided by author and by group (check for repetition, patterns, differences in patterns between groups)	column and row
	Same as before?
	we check for patterns
	An example of this would be useful
Plot differences between consecutive column values in order provided by author and by group (check for repetition, patterns, differences in patterns between groups)	What does this mean?
	we check for patterns and repetitions
Probability of column sequences via simulation and resampling	I don't know what this is
	Useful but has different application and it is operator dependant, particularly if we are going to consider the cumulative sequence.
	What does this mean?
	we don't do this
Test of runs of the same value (e.g. resampling, Wald-Wolfowitz)	What does this mean?
	never heard of
Checks of sequences in decimal places (after deleting integer)	I don't know what this is
	And checks of sequence of integers after deleting decimals.
	What does this mean?

medRxiv preprint doi: https://doi.org/10.1101/2024.03.18.24304479; this version posted March 25, 2024. The copyright holder for this preprint (which was not certified by peer review) is the author/funder, who has granted medRxiv a license to display the preprint in perpetuity.
It is made available under a CC-BY 4.0 International license .

	This is unclear to me? You mean any continuous values?
	we don't do this
Examine relationships between variables for biological plausibility (e.g. by plotting against each other)	this - and several others below - is not phrased as a question
	probably a good idea but we don't do it
Statistical test to compare multivariate correlations between variables between treatment groups (several methods)	What does this mean?
	add the words, "shows unexplained heterogeneity"
	we don't do this
	some example methods and references would help
Plot correlation coefficients for each pair of variables by group using greyscale/ heatmap	Or plot ORs for binary data, e.g. we found an OR of about 30000 for some in a vitamin D paper, where it seemed columns of data had been copied with only 1 or 2 changed
	what pairs?
	never heard of
Compare kurtosis of baseline variables between groups	I don't know what this is
	Also, evidence of truncated distributions?
	What does this mean?
	Not very relevant in my opinion. Randomization should lead to the same kurtosis, but kurtosis sampling fluctuations are known to be extreme, so that very large differences are expected due to randomness.
	we don't do this
Consider whether distribution of variable follows simple but implausible model (such as Normal)	Is a normal distribution implausible for health data?
	Indeed, non-significant normality tests on large samples (>2000) may be indicative of a database generated by software, especially if it is found on several variables.
	You mean it follows it 'too' well?
	yes, basic check
Check repeated measures for interpolation and duplication	I don't know what this is
	never heard of
	I don't know what this one means
Inspect recruitment over	Will you get dates?

medRxiv preprint doi: https://doi.org/10.1101/2024.03.18.24304479; this version posted March 25, 2024. The copyright holder for this preprint (which was not certified by peer review) is the author/funder, who has granted medRxiv a license to display the preprint in perpetuity.
It is made available under a CC-BY 4.0 International license .

calendar time (compare between groups)	we perform thorough checks on dates (T2E data)
Inspect time between participant visits	Why?
	Unclear to me.
	yes, basic check on dates
	What would one be looking for here?
Check visit dates (plausibility of visits on Sundays)	Or Friday in Islamic societies, Saturday in Israel.
	I would change to 'plausibility of visit dates on holidays/weekends'
	as long as dates are collected in local time, or can be set to local time
	Other countries have different weekends, e.g., Egypt weekend is Friday.
	Not a problem in some countries or for some conditions
	Or Fridays, depending on location
	and public holidays
	Rephrase, perhaps: "Are there irregularities in the dates of visit? (for instance, often on Sundays)"
	we check days of randomisation
Plot Chernoff faces for each group	I was not aware that these had any application for diagnosing problematic data
	I don't know what this is
	doesn't seem that helpful
	What does this mean?
	never heard of
	Unclear
	very cool!
Make Star Plots for each group	I am unfamiliar with star plots
	I don't know what this is
	no
	What does this mean?
	again, not familiar with this
	Not sure what that is
	never heard of
	Unclear

medRxiv preprint doi: https://doi.org/10.1101/2024.03.18.24304479; this version posted March 25, 2024. The copyright holder for this preprint (which was not certified by peer review) is the author/funder, who has granted medRxiv a license to display the preprint in perpetuity.
It is made available under a CC-BY 4.0 International license .

	this section contains many items that feel more like a laundry-list than other sections. Maybe organizing it with subsections of what type of fraud or fabrication we are trying to detect would be useful, or what kind of data are available to work with? That might help users choose between the various methods.
Apply neighbourhood clustering method of Wu and Carlsson	I don't know what this is
	not familiar with this
	What does this mean?
	never heard of
	Unclear
	Specifying the objective of doing this (and all of these, actually) would be useful - e.g., "to detect X"
Are data internally consistent?	not sure what this means
	Maybe give an example, for instance dose of drug infused between minimum and maximum rates during a reported time vs the total dose infused.
	This looks vague.
	yup that's what we do in our IPD checks
	How? What checks would be helpful here?
	Unclear
	this question is very general - could we specify a bit more/
Are only a small number of baseline characteristics collected in IPD	not sure this is a sign of fraud, happy to be corrected
	Is this a good or a bad thing?
	if the database analyzed is supposed to be the complete database.
	This looks unimportant.
	Small is less than 4?
	in small trials you will have a small number of baseline characteristics... also you might not have access to the entire dataset due to limited budget (in one IPDMA we could afford to access "buy" only a subset of variables)
Calculate autocorrelation between column values, overall and by group	What does this mean?

medRxiv preprint doi: https://doi.org/10.1101/2024.03.18.24304479; this version posted March 25, 2024. The copyright holder for this preprint (which was not certified by peer review) is the author/funder, who has granted medRxiv a license to display the preprint in perpetuity.
It is made available under a CC-BY 4.0 International license .

Check whether the randomisation sequence consistent with the description in the paper	Also, given knowledge of block sizes, are the ITT group numbers identical, or larger than permitted by blocks?
	yes, basic check

Do you know of any methods not listed here, or do you have any to suggest? Please describe them here if so.

no
No
"Reported information on consent process of trial participants. Willingness of triallists to share such information.<br>Use of paper methods rather than electronic data capture methods for patient reported outcome measures."
No additional thoughts.
Based on some findings which created a stir in my field (detailed here: http://deevybee.blogspot.com/2015/02/editors-behaving-badly.html), we have begun looking at submission to acceptance latency by extracting meta-data from PubMed. Most journals in our sample do not publish both, so we've then moved to emailing journal editors directly. Several have provided dates, but some (notably, The Lancet) have refused. One can then examine the distribution of latency to identify outliers, and use meta-regression or subgroup analysis to examine whether effects for these studies are significantly larger.
"I have additional suggestions for some of the sections, please see below. Some of these may be already encompassed by existing questions:<br><br>*Inspecting research team*<br><br>Given the nature of the study, does author list make sense - i.e. if you have a very simple study (esp.

medRxiv preprint doi: https://doi.org/10.1101/2024.03.18.24304479; this version posted March 25, 2024. The copyright holder for this preprint (which was not certified by peer review) is the author/funder, who has granted medRxiv a license to display the preprint in perpetuity.
It is made available under a CC-BY 4.0 International license .

theoretical/in silico) with dozen authors from different institutions and very diverse expertise. -> captured more generally in criteria for authorship, but this is more specific case<br><br>Do any of the authors have inflated number of publications the topic of which is not universally aligned with this author's expertise.<br><br>Do emails provided for the authors - esp. if institutional - align with stated affiliations.<br><br>*Inspecting publication details*<br><br>Does the speed of consideration appears unusually short (i.e. suspiciously quick time from submission to acceptance).<br><br>*Inspecting results*<br><br>Are statistical tests internally consistent (example: paper reports both p-value and t statistic, but these are not consistent with each other)<br><br>Where original data for graphs provided (usually on request for integrity investigations), can graphs be reproduced. In particular, recapitulation of reported error bars.<br><br>*Inspecting conduct/governance*<br><br>Where ethical approvals provided (usually on request for integrity investigations): do dates on approval align with the description of when the study happened<br><br>Is the procedure of the study aligned with local legislations - this is more for animal research, when legislation can differ significantly between countries, but there is still expectation that what is done, is at least legal in the location where the study happened. Can be important esp. if authors in various countries and laws more permissive not where actual work took place."
A very rough measure (although many other measures are the same) - publications from certain areas of the globe? Or originally published in other languages than english?<br><br>The size of the study (e.g. number of enrollera participants)?

medRxiv preprint doi: https://doi.org/10.1101/2024.03.18.24304479; this version posted March 25, 2024. The copyright holder for this preprint (which was not certified by peer review) is the author/funder, who has granted medRxiv a license to display the preprint in perpetuity.
It is made available under a CC-BY 4.0 International license .

"'- are important features missing from the paper (eg test statistic or degrees of freedom expected but missing; figure expected but missing - it happens)<br>- are there surprisingly few authors - eg a senior professor single-authoring a large experimental study<br>- are data in the publication consistent with the preregistration<br>- if the ethics review number is given, is it consistent with the enrolment start date (for example not ""2021-PQRST"" if enrolment started in in 2020"
"If authors provide an excel spreadsheet, then you could check the meta-data in the sheet, including things like when it was created, by whom, and the number of hours it's been opened. This will not be as useful if the excel is just an export from REDCap or similar.<br><br>Perhaps a mention of the use of REDCap or similar clinical trial management software. I suspect people using these tools are less likely to be fraudulent. "
I would like to suggest if any concern regarding the amount of data collected (the robustness of the trial) and the number of authors and funding reported. Also, to consider the main country from the report of the trial come from.
Check 'information' of file to determine when it was authored and by whom.<br><br>Reorder rows by different column values: sometimes patterns become apparent, which the authors obscure by 'reshuffling' on another column value after fabricating data.<br><br>Nominal variables sometimes have patterns - for instance, I analysed a submission in which the first syllable of names contained repeated sequences.<br><br>The title of the paper should be searched via Google, ResearchGate etc as well as Pubmed.
Are all methods in Weibel et al.,""Identifying and managing problematic trials: a research integrity assessment tool for randomized controlled trials in evidence synthesis"" included?
"Are the results reproducible at all? (if not, this is often used to hide a lot of issues in ambiguity)<br>Multi-center trial comparisons (meta-analysis often results in detecting issues as they are so discrepant from everything else)<br>Could compare hash files of the data provided as real (i.e., forensic investigation of the data origins) - this requires a bit more but there is value in storing these to detect changes over time in a secure manenr."
Check that when the dataset is ordered by participant ID or randomisation timestamp, the N+1th participant has the same condition as the Nth 1/k of the time, where there are k conditions. If the condition assignment has been fabricated "by hand", the condition will often change too frequently as

medRxiv preprint doi: https://doi.org/10.1101/2024.03.18.24304479; this version posted March 25, 2024. The copyright holder for this preprint (which was not certified by peer review) is the author/funder, who has granted medRxiv a license to display the preprint in perpetuity.
It is made available under a CC-BY 4.0 International license .

the faker tries to avoid "excessively long identical sequences".
1- A frequency distribution table or graph for baseline characteristics can give a good impression of the quality of randomisation at first glance.<br>2- The plausibility of the number of duplicated values (cases) across numeric variables within a data set.<br>3- Test whether a variable is a subset of a second variable within a data set.<br>4- Test binary data for consistency if their mean and SD are given.<br>5- An interaction test to assess the subgroup homogeneity to detect data manipulation to achieve implausible consistency (the p-value of the Tarone-adjusted Breslow-Day test).<br>6- Check whether withdrawal and loss to follow-up in multiple trials by the same author are consistent with the expected (random) binomial distribution (check Carlisle 2012 and Bolland 2020).<br>7- For trials that did not report mean but reported median, the Box-Cox method of McGrath et al. (2020) and the MLN method of Cai et al. (2021) can be used to convert the median (IQR or range) to mean (SD). The converted mean (SD) can be used for regular checks of summary stats. Whether the Box-Cox method of McGrath et al. (2020) and the MLN method of Cai et al. (2021) are compelling needs to be evaluated.
Consider the legitimacy of the journal where the study has been published (i.e a predatory journal or publisher)<br><br>Other papers which may be covered already in the inspecting results but just in case:<br>https://pubmed.ncbi.nlm.nih.gov/28412468/<br>https://journals.sagepub.com/doi/full/10.1177/1948550616673876<br>https://www.semanticscholar.org/paper/Using-Statistics-from-Binary-Variables-to-Detect-Schumm-Crawford/52008d10f8f942672cb4d1334978e5299bd213b4
Not sure if I missed it. But what about data fields missing from the IPD i.e. the paper reports data subgrouped by sex but sex is not available in the IPD

If you have any comments about this survey, or about this topic more generally, please add them here.

It seems it would be very difficult to get some of the information to answer these questions.
Signalling questions or prompts for some of these questions will be needed to help guide users of the tool to look at the right bits of information.
It would be good to list simple first line tests for integrity to start with, not necessarily involving much statistical knowledge, to use before moving to more complex methods.

medRxiv preprint doi: https://doi.org/10.1101/2024.03.18.24304479; this version posted March 25, 2024. The copyright holder for this preprint (which was not certified by peer review) is the author/funder, who has granted medRxiv a license to display the preprint in perpetuity.
It is made available under a CC-BY 4.0 International license .

Something else I've noticed in potentially problematic studies are samples that appear to be connected across studies even though articles fail to indicate they are connected or even imply they are separate samples. In these cases, the investigator has published what appear to be multiple separate RCTs of a method, but in fact the sample is the same and the data has simply been re-analyzed with a few participants removed. I can't quite say what tips off when this is happening- sample sizes that are quite close, sample means that are very similar?
No comments. This looks like a great resource - best of luck with the project!
"Something that systematic reviewers would appreciate is a tool that is easy to use and possibly not too time intensive.<br>A thought is to have both a short version of a research integrity tool as well as a longer version. Or implementing it at different parts of the SR process (e.g. basic first screen and then a more intense assessment further to the finished process, or as a part of a sensitivitet analysis)" It would be good to work with ScreenIT on this. They are a great group and very collaborative in my experience.
Very complete. Thanks for having me here.
We could do with a searchable record of authors who have submitted unpublished false data (similar to the RetractionWatch database of published papers), populated by verified journal editors. Most of the false trials I identified have been published elsewhere (often with major changes); there is no mechanism to reduce this happening. I built a sandcastle against the tide.
Is it worth considering conflict of interest (declared or not) - often a source of considerable bias which is certainly problematic, clearly uncontrolled. Depends on what one considers a problematic trial - is it one that cripples reproducibility? Barbara
"Lots falls and stands based on the details of implementing. Lots of these methods are theoretically feasible, but their in-practice validity is hard with low prevalence of fabrication. If applied often and without prior suspicion, this can lead to many false positives and reduces trust in the institutions providing these assessments. It would be best to take a two-staged approach - if you suspect an author/group based on a paper, move to other papers to see whether those too have problems.<br><br>What also works particularly well is comparing studies of a similar nature - genuine and ungenuine data will be often easier to sort out. If it's harder to sort out, the effect of the ungenuine data (if present) will also be smaller and the risk is smaller)."
Very comprehensive - well done to the team!
This is fairy comprehensive. Clustering methods may be useful but I haven't used them. In the only retraction I've been involved in, the individual patient data was so poor that it didn't merit any formal statistical methods: the rows in the dataset did not correspond to individual patients (it seemed that

medRxiv preprint doi: https://doi.org/10.1101/2024.03.18.24304479; this version posted March 25, 2024. The copyright holder for this preprint (which was not certified by peer review) is the author/funder, who has granted medRxiv a license to display the preprint in perpetuity.
It is made available under a CC-BY 4.0 International license .

each column of data was constructed individually: pairwise scatterplots were sufficient to detect the problem). Also, the randomisation sheet was just a set of random numbers - but no treatments listed beside them. I don't think the full gambit of methods described above are necessary for every case. I'll e-mail you my checklist for new submissions - it's very similar to many lists out there - but it doesn't go into methods for individual patient data assessment.
Some of the questions in this survey are very technical or difficult to understand. Have the people who suggested them run the suggested tests against IPD from their own trials?
This is really important work!
"V useful list.<br>I've thought for ages that we need a Uni to set up a MSc coures to train people in methods of fraud detection. Maybe Manchester could be persuaded to do it.<br><br>I'm now retired and doing this kind of thing on an amateur basis. For instance, I have reservations about a study by XXXX that I've flagged on PubPeer - I haven't tried to get the data, but the study seemed particularly problematic in terms of plausibility of time scale etc. https://pubpeer.com/publications/888BF5CC8DBCD2B080DC4189AA604D. I've tried to get confirmation from the Ethics organisation in iran but not had any success.<br><br>One other thing: this task would be made MUCH easier if all ethics approvals were made open. There seems huge reluctance to do that.<br><br>XXXXXX"
This seems a very exhaustive and comprehensive list.
"As scientific misconduct is a continuum, it may not be easy to distinguish between scientific misconduct, poor methodology, poor reporting and major protocol deviations, with possible overlap between these notions.<br><br>For instance, I found an article where the methods section specified that some costy and hard-to-retreive data was collected (SARS-Cov-2 RT-PCR every week in 10000 asymptomatic indian healthy volounteers), but there were no result related to these data in the Results section, suggesting that it was actually never done. Strangely, there extensive data with poor clinical relevance was reported (serological responses) while the most important results (COVID-19 symptomatic and asymptomatic infections) had very little reporting in the Results section.

medRxiv preprint doi: https://doi.org/10.1101/2024.03.18.24304479; this version posted March 25, 2024. The copyright holder for this preprint (which was not certified by peer review) is the author/funder, who has granted medRxiv a license to display the preprint in perpetuity.
It is made available under a CC-BY 4.0 International license .

I suspect that there were major deviations from the original protocol and that most of the clinically relevant data could not be used, so that authors reported as few data as possible on that. However, they reported nothing on these problems and even claimed to have very few lost to follow-up. To what degree is there an intentional hiding of these problems?<br><br>Where does start the poor reporting? Where does start the scientific misconduct? Is poor reporting a scientific misconduct?<br><br>"
"Wow there are some methods there I never heard of.<br>Great work!"
Are you working with XXXXXX on this?
"A technical note, the space to provide comments is very limited.<br><br>On the topic, most of listed activities are sensible but from the practical point of view not sure how one would be able to perform all the checks within a reasonable time when we already know that syst. reviews take too much time to complete.<br><br>As for the IPD checks, haven't heard about 60% of listed methods<br>"
"Just adding my response here per our meeting to note my software in development, that will permit semi-automatic assessments of some of the items in this list (starting with the distribution of baseline characteristics from RCTs). I hope to have it online in the next month.<br><br>- XXXXXX"
I would have like to be able to score the items as to how important they were.
Thanks for the opportunity to comment. My first reaction is that this is a great list. My second reaction is that there are a lot of items to check. Would the average reviewer have the expertise and time to go through this list? Could the list be shortened or prioritised (e.g., the retraction question might be the first one, in which case the reviewer wouldn't need to proceed)? Are there opportunities to automate some of these steps (e.g., plagiarism, attaching a link of retractions connected to the paper or author team)?<br>All the best
I think methods should be divided in those that can be used more for screening (so high sensitivity, even with the risk of lots of false positives) and those that are more diagnostic.

medRxiv preprint doi: https://doi.org/10.1101/2024.03.18.24304479; this version posted March 25, 2024. The copyright holder for this preprint (which was not certified by peer review) is the author/funder, who has granted medRxiv a license to display the preprint in perpetuity.
It is made available under a CC-BY 4.0 International license .

Many aspects relevant for the research integrity assessment (e.g. assessing proper randomization, author contributorship, funding details, ethics...) can only be checked if sufficient details are reported in a publication, protocol or registry record. Problematic studies often do not report in sufficient detail on aspects relevant for assessment of RI. Thus, I believe that insufficient reporting or non-reporting itself on several relevant RI aspects is a matter of ignoring research integrity and should be a red flag.
It seems like it would be useful to further organise and categorise the list as presented here. e.g. Inspecting results and inspecting data should juxtaposed; different approaches to testing for fabricated data (digit analysis vs multivariate distributions) should be grouped; etc.
It might be useful to review the reported cases noted above and other more, e.g. retracted papers, famous investigation reports (https://www.nature.com/articles/d41586-021-00733-5) and propose other methods to inspect the problems?
This is really useful and I am so eager to see the final product, and learn more about the various methods you have mentioned. If I can be helpful in any way I'd love to remain involved. My stake is as a consumer of the medical literature and critical appraisal enthusiast responsible for teaching clinicians how to read the literature, which includes knowing how to detect fraud. In case there is not space for this later, my name is XXXXXXX. Thanks for doing this important work!
"Do not make the list too long. Aks yourself the question 'are the data true' and in doubt, ask for original data?<br><br>PS: I really like the input of Jack and his team in the field of research integrity; you are making a difference!!"
This is a very comprehensive list of identifiers, much more than I have thought of previously.
"This is an excellent list and I have nothing much to add (I have limited statistical expertise) except under 'inspecting the research team:<br>Are there authors on the paper from the country from which the data was collected (i.e to counter safari/parachute research).<br>"
Diagnostic yield versus time taken to test. Major impediment is probably getting access to the raw data, and some simple tests (such as time to get the data) may end up performing as well as more complex ones. I think this is a structural problem with the medical literature, and is solvable to a large extent by deconstructing study information into freely accessible bits which are commented on by content experts for that piece (much would still be with statisticians) and then the linked red flag appears in the public domain.

medRxiv preprint doi: https://doi.org/10.1101/2024.03.18.24304479; this version posted March 25, 2024. The copyright holder for this preprint (which was not certified by peer review) is the author/funder, who has granted medRxiv a license to display the preprint in perpetuity.
It is made available under a CC-BY 4.0 International license .

Section B. Updated list of checks for problematic studies

Checks are arranged in five domains. Numbers in brackets/ parentheses are citation in main text.

Domain 1: Inspecting the results in the paper (28 checks)

Check
Is the number of participant withdrawals compatible with the disease, age and timeline?(7)
Are subgroup means incompatible with those for the whole cohort?(7)
Are the reported summary data compatible with the reported range?(7)
Are the summary outcome data identical across study groups?(7)
Are there any discrepancies between data reported in figures, tables and text?(7)
Are statistical test results compatible with reported data?(7, 15)
Are any baseline data implausible with respect to magnitude or variance? (7)
Are any outcome data, including estimated treatment effects, implausible?(16)
Are any of the baseline data excessively similar between randomized groups?(17, 18)
Are any of the baseline data excessively different between randomised groups? (17, 18)
Are there any discrepancies between the values for percentage and absolute change?(7)
Are there any discrepancies between reported data and participant inclusion criteria?(7)
Are the variances in biological variables surprisingly consistent over time?(7)
Are correct units reported?(7)
Are numbers of participants correct and consistent throughout the publication?(7)
Are calculations of proportions and percentages correct?(7)
Are results internally consistent?(7)
Are non-first digits compatible with a genuine measurement process?(19)
Are the variances of integer data possible?(20)
Are the means of integer data possible?(21)
Are data simulated from reported summary statistics plausible?(22)
Are differences in variances in baseline variables between randomised groups plausible(using

medRxiv preprint doi: https://doi.org/10.1101/2024.03.18.24304479; this version posted March 25, 2024. The copyright holder for this preprint (which was not certified by peer review) is the author/funder, who has granted medRxiv a license to display the preprint in perpetuity.
It is made available under a CC-BY 4.0 International license .

summary data)?(23, 24)
Are coefficients of variation plausible?
Is the amount of missing data plausible?(25)
Are the results substantially divergent from the results of multiple other studies in meta-analysis(26)
Is there heterogeneity across studies in degree of imbalance in baseline characteristics (in meta-analysis)(27)
Are statistical tests internally consistent? (example: paper reports both p-value and t statistic, but these are not consistent with each other) [origin: from current survey]
Are important features missing from the paper? [origin: from current survey]

Domain 2: Inspecting the research team and their work (19 checks)

Have the data been published elsewhere by the research team in an illegitimate fashion?(7)
Is any duplicate reporting acknowledged or explained?(7)
Are duplicate-reported data consistent between publications?(7)
Are relevant methods consistent between publications?(7)
Is there evidence of duplication of figures?(7)
Does consideration of other studies from members of the research team highlight causes for concern?(6)
Is the distribution of non-first digits in manuscripts from one author compatible with a genuine measurement process?(28)
Is the standard deviation of summary statistics in multiple studies by same authors plausible (when compared to simulated or bootstrapped data?)(29)
Do all authors meet criteria for authorship?(7)
Are contributorship statements present?(7)
Are contributorship statements complete?(7)
Is authorship of related papers consistent?(7)
Can co-authors attest to the reliability of the paper?(7)
Have other studies from the author team been retracted, or do they have expressions of concern, relevant post-publication amendment, or critical Retraction Watch or PubPeer

medRxiv preprint doi: https://doi.org/10.1101/2024.03.18.24304479; this version posted March 25, 2024. The copyright holder for this preprint (which was not certified by peer review) is the author/funder, who has granted medRxiv a license to display the preprint in perpetuity.
It is made available under a CC-BY 4.0 International license .

comment?(6)
Are the authors on staff of institutions they list?(6)
Do any authors have a professorial title but no other publications on PubMed?(6)
Does the statistics methods section use generic language, suggesting lack of expert statistical input?(6)
Are withdrawal and loss to follow-up in multiple trials by the same author consistent with the expected (random) binomial distribution? [origin: from current survey]
Given the nature of the study, does the author list make sense? - i.e. does a simple study have dozens of authors from different institutions and with diverse expertise. [origin: from current survey]

Domain 3: Inspecting conduct, governance and transparency (21 checks)

Is the volume of work reported by research group plausible, including that indicated by concurrent studies from the same group?(7)
Is the reported staffing adequate for the study conduct as reported?(7)
Is the recruitment of participants plausible within the stated time frame for the research?(7)
Is the recruitment of participants plausible considering the epidemiology of the disease in the area of the study location?(7)
Is the interval between study completion and manuscript submission plausible?(7)
Could the study plausibly be completed as described?(7)
Are the study methods plausible, at the location specified?(7)
Are the locations where the research took place specified, and is this information plausible?(7)
Is a funding source reported?(7)
Has the study been prospectively registered?(7)
Are details such as dates and study methods in the publication consistent with those in the registration documents?(7)
Is there evidence that the work has been approved by a specific, recognized committee? (ethics)(7)
Are there any concerns about unethical practice?(7)

medRxiv preprint doi: https://doi.org/10.1101/2024.03.18.24304479; this version posted March 25, 2024. The copyright holder for this preprint (which was not certified by peer review) is the author/funder, who has granted medRxiv a license to display the preprint in perpetuity.
It is made available under a CC-BY 4.0 International license .

Is the grant funding number identical to the number in unrelated studies?(6)
Are the data publically available?(6)
Do the authors agree to share individual participant data?(6)
Are additional patient data recorded in patient case records beyond what is reported in the paper?(6)
Do authors cooperate with requests for information? [author team suggestion]
Do authors provide satisfactory responses to requests? [author team suggestion]
In which country was the study conducted? [origin: from the current survey]
Is the procedure of the study aligned with local legislations? [origin: from the current survey]

Domain 4: Inspecting text and publication details (8 checks)

Is there evidence of copied work, such as duplicated or partially duplicated tables?(7)
Is there evidence of text reuse (cutting and pasting text between papers), including text that is inconsistent with the study?(7, 30-34)
Are there typographical errors?(7)
Is there evidence of automatically-generated text?(35)
Has the study been retracted or does it have an expression of concern, a relevant post-publication amendment, a critical Retraction Watch or PubPeer comment or has been previously excluded from a systematic review?(6)
Was the study published in a journal from a list of predatory/ low quality journals?(6)
Is there evidence of manipulation or duplication of images?(7)
Was the time between submission to acceptance reasonable? [origin: from current survey]

Domain 5: Inspecting individual participant data (40 checks)

Compare leading digits in individual participant data to Benford's Law(28)
Compare non-first digits in individual participant data to expected (36)
Compare distribution of leading digits in individual participant data between randomised arms [author team suggestion]
Compare distribution of non-first digits in individual participant data between randomised

medRxiv preprint doi: https://doi.org/10.1101/2024.03.18.24304479; this version posted March 25, 2024. The copyright holder for this preprint (which was not certified by peer review) is the author/funder, who has granted medRxiv a license to display the preprint in perpetuity.
It is made available under a CC-BY 4.0 International license .

arms(36)
Comparing multiple versions of a spreadsheet containing study data for consistency(9)
Examining spreadsheet for formulae used to fabricate data(9)
Change global format to 'general' in Excel - calculated values display long strings of numbers to right of decimal place, fabricated values may not(9)
Can the results in the paper be reproduced from the underlying dataset?(9)
Statistical test to compare variances in baseline variables between groups using IPD (Levene, Brown-Forsythe)(36-38)
Identifying inliers using singular value decomposition(39)
Identifying inliers using Mahalanobis distance(10)
Colour code values in Excel spreadsheet to highlight outlying values, patterns and repetition(40)
Plot column values in order provided by author and by group (check for repetition, patterns, differences in patterns between groups)(40)
Plot differences between consecutive column values in order provided by author and by group (check for repetition, patterns, differences in patterns between groups)(40)
Probability of column sequences via simulation and resampling(40)
Test of runs of the same value (e.g. resampling, Wald-Wolfowitz)(41)
Checks of sequences in decimal places (after deleting integer)(40)
Examine relationships between variables for biological plausibility (e.g. by plotting against each other)(10)
Statistical test to compare multivariate correlations between variables between treatment groups (10)
Plot correlation coefficients for each pair of variables by group using greyscale/ heatmap(14)
Compare kurtosis of baseline variables between groups(10)
Consider whether distribution of variable follows simple but implausible model (such as Normal)(10)
Check repeated measures for interpolation and duplication(10)
Inspect recruitment over calendar time (compare between groups)(10)
Inspect time between participant visits(10)

medRxiv preprint doi: https://doi.org/10.1101/2024.03.18.24304479; this version posted March 25, 2024. The copyright holder for this preprint (which was not certified by peer review) is the author/funder, who has granted medRxiv a license to display the preprint in perpetuity.
It is made available under a CC-BY 4.0 International license .

Check visit dates (plausibility of visits on Sundays)(10)
Plot Chernoff faces for each group(10, 14)
Make Star Plots for each group(10, 14)
Apply neighbourhood clustering method(42)
Are data internally consistent? [author team suggestion]
Are only a small number of baseline characteristics collected in IPD(6)
Calculate autocorrelation between column values, overall and by group [author team suggestion]
Check whether the randomisation sequence consistent with the description in the paper [author team suggestion]
If authors provide an excel spreadsheet, then you could check the meta-data in the sheet, including things like when it was created, by whom, and the number of hours it's been opened. This will not be as useful if the excel is just an export from REDCap or similar. [origin: from current survey]
Reorder rows by different column values: sometimes patterns become apparent, which the authors obscure by 'reshuffling' on another column value after fabricating data. [origin: from current survey]
Check that when the dataset is ordered by participant ID or randomisation timestamp, the N+1th participant has the same condition as the Nth 1/k of the time, where there are k conditions. If the condition assignment has been fabricated "by hand", the condition will often change too frequently as the faker tries to avoid "excessively long identical sequences. [origin: from current survey]
Data fields missing from the IPD i.e. the paper reports data sub-grouped by sex but sex is not available in the IPD. [origin: from current survey]
Test whether a variable is a subset of a second variable within a data set. [origin: from current survey]
The plausibility of the number of duplicated values (cases) across numeric variables within a data set. [origin: from current survey]
An interaction test to assess the subgroup homogeneity to detect data manipulation to achieve implausible consistency (the p-value of the Tarone-adjusted Breslow-Day test). [origin: from current survey]

medRxiv preprint doi: https://doi.org/10.1101/2024.03.18.24304479; this version posted March 25, 2024. The copyright holder for this preprint (which was not certified by peer review) is the author/funder, who has granted medRxiv a license to display the preprint in perpetuity.
It is made available under a CC-BY 4.0 International license .

Section C. Items excluded from the survey. Numbers I brackets/ parentheses are citation in main document.

Excluded check	Origin	Reason for exclusion
Is the number of participant deaths compatible with the disease, age and timeline?	Review of papers included in scoping review (7)	Covered by checks relating to plausibility of study outcomes
Are any data impossible?	Review of papers included in scoping review (7)	Covered by several more specific checks
Are any of the outcome data unexpected outliers?	Review of papers included in scoping review (7)	Covered by *Are any outcome data, including estimated treatment effects, implausible?*
Are any data outside the expected range for sex, age or disease?	Review of papers included in scoping review (7)	Covered by several more specific checks
Are the results of statistical testing internally consistent and plausible?	Review of papers included in scoping review (7)	Covered by *Are statistical test results compatible with reported data?* [Note: was subsequently added to the list in response to the survey]
How many data are duplicate reported?	Review of papers included in scoping review (7)	Covered by checks relating to duplicate reporting.
Are other data errors present?	Review of papers included in scoping review (7)	Considered too vague, covered by other more specific items.
Investigating all publications of one author	Scoping review	Covered by several more specific items (what and how to check the work of the author)

medRxiv preprint doi: https://doi.org/10.1101/2024.03.18.24304479; this version posted March 25, 2024. The copyright holder for this preprint (which was not certified by peer review) is the author/funder, who has granted medRxiv a license to display the preprint in perpetuity.
It is made available under a CC-BY 4.0 International license .

Inspect variances over time	Review of papers included in the scoping review (10)	Covered by *Are the variances in biological variables surprisingly consistent over time?*
Was a reporting checklist used?	Qualitative study	Outside of scope (reporting quality)
Do the numbers of animals purchased and housed align with numbers in the publication?	Review of papers included in scoping review (7)	Could not apply to human studies
Have the correct analyses been undertaken and reported?	Review of papers included in scoping review (7)	Outside of scope (study quality)
Is there evidence of poor methodology, including: missing data, inappropriate data handling, 'P-hacking': biased or selective analyses that promote fragile results, other unacknowledged multiple testing.	Review of papers included in scoping review (7)	Outside of scope (risk of bias)
Is there outcome switching — that is, do the analysis and discussion focus on measures other than those specified in registered analysis plans?	Review of papers included in scoping review (7)	Outside of scope (risk of bias)
Have not included specific methods for statistical monitoring/ fraud detection of multicentre trials using IPD (trying to detect fabrication at one site by comparing to others)	Scoping review (e.g. (8))	Not applicable outside of central trial monitoring context
Checking grant applications for inconsistencies in reported preliminary results	Review of papers included in scoping review (9)	Proposed in context of institutional integrity investigations – relies on

medRxiv preprint doi: https://doi.org/10.1101/2024.03.18.24304479; this version posted March 25, 2024. The copyright holder for this preprint (which was not certified by peer review) is the author/funder, who has granted medRxiv a license to display the preprint in perpetuity. It is made available under a CC-BY 4.0 International license .

		access to grant applications.

medRxiv preprint doi: https://doi.org/10.1101/2024.03.18.24304479; this version posted March 25, 2024. The copyright holder for this preprint (which was not certified by peer review) is the author/funder, who has granted medRxiv a license to display the preprint in perpetuity.
It is made available under a CC-BY 4.0 International license .
